# Developing a Chromatographic ^99m^Tc Generator Based on Mesoporous Alumina for Industrial Radiotracer Applications: A Potential New Generation Sorbent for Using Low-Specific-Activity ^99^Mo

**DOI:** 10.3390/molecules27175667

**Published:** 2022-09-02

**Authors:** Mohamed F. Nawar, Alaa F. El-Daoushy, Ahmed Ashry, Andreas Türler

**Affiliations:** 1 Department of Chemistry, Biochemistry and Pharmaceutical Sciences, Faculty of Science, University of Bern, Freiestrasse 3, CH-3012 Bern, Switzerland; 2 Radioactive Isotopes and Generators Department, Hot Laboratories Center, Egyptian Atomic Energy Authority, Cairo 13759, Egypt; 3 Radiation Protection and Civil Defense Department, Nuclear Research Center, Egyptian Atomic Energy Authority, Cairo 13759, Egypt

**Keywords:** LSA ^99^Mo, ^99^Tc generators, industrial radiotracers, mesoporous alumina

## Abstract

The commercial low-pressure column chromatographic ^99^Mo/^99m^Tc generator represents a reliable source of onsite, ready-to-use ^99m^Tc for industrial applications. These generators use fission-produced ^99^Mo of high specific activity, posing serious production challenges and raising proliferation concerns. Therefore, many concepts are aimed at using low-specific-activity (LSA) ^99^Mo. Nonetheless, the main roadblock is the low sorption capacity of the used alumina (Al_2_O_3_). This study investigates the feasibility of using commercial alumina incorporated with LSA ^99^Mo to develop a useful ^99^Mo/^99m^Tc generator for industrial radiotracer applications. First, the adsorption profiles of some commercial alumina sorbents for LSA ^99^Mo were tested under different experimental conditions. Then, the potential materials to develop a ^99^Mo/^99m^Tc generator were selected and evaluated regarding elution yield of ^99m^Tc and purity. Among the sorbents investigated in this study, mesoporous alumina (SA-517747) presented a unique sorption-elution profile. It demonstrated a high equilibrium and dynamic sorption capacity of 148 ± 8 and 108 ± 6 mg Mo/g. Furthermore, ^99m^Tc was eluted with high yield and adequate chemical, radiochemical, and radionuclidic purity. Therefore, this approach provides an efficient and cost-effective way to supply onsite ^99m^Tc for radiotracer applications independent of fission-produced ^99^Mo technology.

## 1. Introduction

Short-lived radionuclides have proved their crucial role in developing different industrial applications [1,2,3,4]. Their contribution helps to provide effective malfunction detection and process optimization. Accordingly, this reduces production costs, enhances process efficiency, and improves product quality [2,3,5]. Notably, ^99m^Tc received considerable attention in multi-disciplinary fields due to its accessible availability and favorable nuclear properties, such as its short half-life of 6.01 h and the emission of low-energetic photons (140 keV) [6,7,8,9,10,11,12,13]. ^99m^Tc is widely available from ^99^Mo/^99m^Tc radionuclide generators. These generators are based on retaining the parent, ^99^Mo, and then the radioactive-decay-generated daughter, ^99m^Tc, can be periodically collected using an isotonic saline solution as an eluent at desired time slots [10,14,15].

It is possible to obtain ^99^Mo in two different product qualities; high- and low-specific-activity products. High-specific-activity ^99^Mo is produced from the neutron-induced uranium fission method. This approach is the most widely used one for large-scale ^99^Mo supply. Over 95% of all ^99^Mo used for the production of ^99m^Tc is available from the fission of highly enriched ^235^U targets in nuclear reactors. Nevertheless, the application of this technology faces inherent critical difficulties. For instance, it is accompanied by severe proliferation concerns. Moreover, the separation and purification of ^99^Mo from fission products is a sophisticated process and calls for large and complex infrastructures, professional technical skills, and well-equipped laboratories. Furthermore, it involves generating massive quantities of radioactive waste [16,17,18]. Therefore, only a few centers worldwide can conduct this task [18,19,20,21]. These difficulties are reflected in the need for long-term capital investments and expensive operating expenditures. However, the generated ^99^Mo is essentially free of stable Mo isotopes and therefore exhibits a very high specific activity of >370 TBq/g (>10,000 Ci/g). In contrast, LSA ^99^Mo is generated either by irradiation of enriched ^98^Mo with epithermal neutrons in a nuclear reactor, or by photonuclear reactions on enriched ^100^Mo targets. Typical specific activities of LSA ^99^Mo are of the order of about 370 GBq/g (≈10 Ci/g), about a factor of 1000 lower.

In order to minimize the dependence on fission-produced ^99^Mo, different ^99m^Tc generator production strategies have been explored and developed over the last few decades by using LSA ^99^Mo. These technologies include sublimation, electrochemical separation, solvent extraction, supported liquid membrane (SLM), and column chromatographic approaches [22,23,24]. Nevertheless, the column chromatography-based generator has attracted considerable interest as a reliable source to supply onsite, ready-to-use ^99m^Tc radionuclide [25,26]. Here, due to its convincing performance as a column material, conventional alumina is loaded with low-specific-activity ^99^Mo. However, based on the fact that conventional alumina possesses distinctly limited sorption capacity (2–20 mg Mo/g) [24], the effective practical implementation of this technology suffered from inherent obstructions. These limitations include the vital need for significant amounts of alumina to build a ^99m^Tc radionuclide generator with a suitable radioactivity level of at least ≈37 GBq (≈1 Ci). Consequently, this results in eluting ^99m^Tc in a relatively large volume with a very low radioactive concentration (RAC), which is accompanied by tedious post-elution concentration steps [20,27]. Furthermore, because of the fact that the sorbent material is the heart of column chromatographic generators, these critical limitations substantially restrict the applicability of conventional alumina in industrial generator systems. Accordingly, they have prompted the need to search for alternate types of sorbents.

Many recent studies have focused on developing a new generation of alumina that possesses a high Mo sorption capacity to efficiently utilize LSA ^99^Mo. These materials are fabricated based on nanotechnology. The use of advanced nano-alumina has gained growing interest in terms of developing ^99m^Tc radionuclide generators [19]. In this context, the utilization of advanced alumina sorbents is an exciting proposition due to their improved properties [28]. This class of alumina possesses appreciable adsorption capacity and unique performance in loading ^99^Mo of low specific activity and the elution of ^99m^Tc. In contrast to traditional sorbents, this new generation of alumina sorbents has large surface-to-volume ratios, enhanced porosity, improved surface reactivity, and significant radiation resistance and chemical stability [29]. Therefore, they show high sorption efficiency and selectivity [14].

This work aims to evaluate the applicability of some commercially available alumina to develop a ^99^Mo/^99m^Tc generator for industrial applications based on LSA ^99^Mo. To reach this goal, the sorption profiles of some selected alumina sorbents were tested for LSA ^99^Mo under different experimental conditions of solution pH, initial Mo concentration, and temperature. Moreover, the feasibility of a ^99^Mo/^99m^Tc generator was demonstrated. Eventually, the elution performances of potential sorbents regarding ^99m^Tc yield and purity were investigated.

## 2. Results and Discussion

### 2.1. Effect of Solution pH

**Figure 1:**
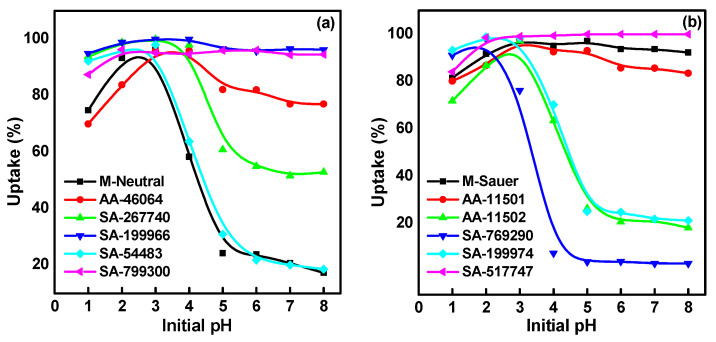

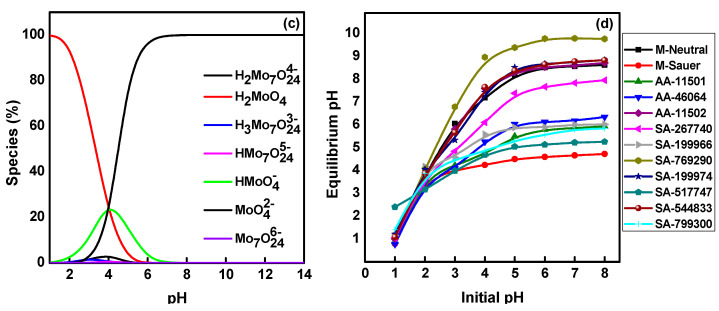
Effect of initial pH (**a**,**b**) on the uptake of CA-^99^Mo on different alumina adsorbents (C_o_ = 50 mg/L, V/m = 100 mL/g, and temperature = 25 ± 1 ^o^C), (**c**) speciation of molybdenum, and (**d**) variation of the equilibrium pH values.

Batch experiments were conducted to verify the usefulness of some commercial alumina for CA-^99^Mo sorption from aqueous solutions. In order to design a successful sorption process, investigating the optimum pH is of crucial concern. The solution pH mainly governs the sorption behavior, as it controls the existing Mo species in the solution and the degree of charge that appears on the sorbent surface [30]. To demonstrate the influence of solution pH on the efficiency of the sorption process, equilibrium studies were conducted at a pH ranging from (1 to 8). The results presented in Figure 1a,b display the uptake of CA-^99^Mo on different commercial alumina as a function of solution pH. Optimal uptake values are observed at an initial pH of around 3. Beyond this value, it can be seen that Mo uptake starts either to decrease very slightly (almost consistent) for M-Sauer, AA-11501, AA-46064, SA-199966, SA-517747 and SA-799300, or sharply, in the case of M-Neutral, AA-11502, SA-267740, SA-769290, SA-199974 and SA-544833. This behavior can be explained based on determining the surface charge of the solid phase and the distribution of the molybdate species in the solution.

On the one hand, the isoelectric point (pH_IEP_) of alumina sorbents varies from (pH 4–6.5), as reported in the literature [31,32]. Consequently, the surfaces of the sorbents carry a positive charge at pH < 4–6.5 and are negatively charged at pH > 4–6.5. On the other hand, the distribution of Mo species at different solution pH values was investigated using the PHREEQC software (version 3) (Figure 1c). At low pH values, different molybdenum anions and polyanions may exist due to the polymerization of the monomeric molybdate anions, MoO_4_^2−^. Molybdate species of MoO_4_^2−^, [Mo_7_O_24_]^6−^, and [Mo_8_O_26_]^4−^ are the most predominant species in this region. These polyanions have higher molybdenum content. Therefore, an electrostatic attraction between negatively charged molybdate anions and positively charged alumina surfaces occurs [33].

The slight and sharp decrease in the uptake affinity of the sorbents can also be assumed based on the isoelectric point (pH_IEP_) of the studied alumina sorbents and the equilibrium pH values (Figure 1d). The sorbents with equilibrium pH values below or within (4–6.5) show a slight decrease in the uptake values. Meanwhile, those with an equilibrium pH > 6.5 (surpassing pH_IEP_) show a sharp decrease in their uptake behavior.

According to the obtained results, we selected seven materials that showed higher sorption of CA-^99^Mo for subsequent investigations. These adsorbents were M-Sauer, AA-11501, SA-267740, SA-199966, SA-517747, SA-544833, and SA-799300.

### 2.2. Thermodynamic Studies

The amount of sorbed CA-^99^Mo was examined as a function of reaction temperature (T). The thermodynamic parameters include Gibbs free energy ΔG° (kJ/mol), standard enthalpy change ΔH° (kJ/mol), and standard entropy change ΔS° (J/mol∙k). These were investigated at different temperatures (298, 313, 323, and 333 K) using Equations (1) and (2) [34,35] and are summarized in Table 1:

(1) $$\Delta G{}^{\circ}=-{\mathrm{RTlnK}}_{d}$$

(2) $$\ln K\;=\frac{\Delta S{}^{\circ}}{R}-\frac{\Delta H{}^{\circ}}{\mathrm{RT}}\;$$

where R is the universal gas constant (8.314 J/mol∙k), T is the absolute temperature (K), and K_d_ (mL/g) is the distribution coefficient.

**Figure 2:**
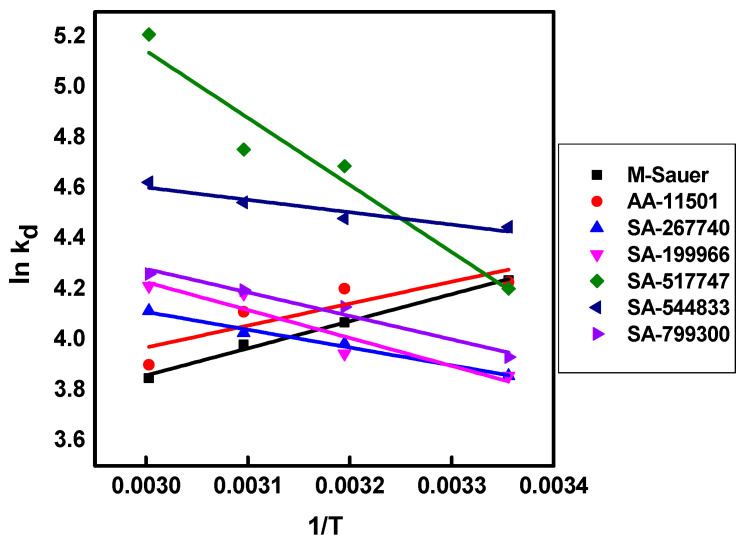
Van’t Hoff plot for the sorption of CA-^99^Mo on different alumina adsorbents.

Linear plots of ln K_d_ versus (1/T) are deployed and presented in Figure 2. The calculated ΔG° values at each temperature for all sorbents are ΔG° < 0, which indicates that the sorption processes of CA-^99^Mo were spontaneous in nature and all the reactions were feasible. The values of ΔG° decrease with increasing temperature, implying that the more the temperature increases, the more the spontaneity degree can be improved. In addition, since the Gibbs free energy lies between −20 < ΔG° < 0, this indicates the occurrence of a physisorption process [36]. The values of ΔS° are positive, which states that random sorption occurs at all alumina adsorbents and Mo(VI) interfaces. The values of ΔH° are negative (ΔH° < 0) for both M-Sauer and AA-11501 adsorbents, implying that Mo(VI) adsorption at their surfaces is exothermic [36,37]. Meanwhile, for SA-267740, SA-199966, SA-517747, SA-544833, and SA-799300 adsorbents, the change in enthalpy (ΔH°) is positive (ΔH° > 0), suggesting that the adsorption of Mo(VI) at their surfaces is endothermic [38].

### 2.3. Adsorption Isotherms

Generally, the ion sorption mechanism for solids can occur either through chemical bond formation (chemisorption) due to the formation of an inner-sphere surface complex or through electrostatic attraction that mainly results from the formation of an outer-sphere surface complex. Moreover, the reactions with solid particles may involve the penetration of the sorbent material or include the formation of precipitates on the adsorbate surface (usually time-dependent). Sorption can be described by empirical sorption isotherms, where the relationship between the concentration of solute in solution (C_e_) and the concentration of solute adsorbed on the surface of adsorbent q_e_ is presented as an X-Y graph. Several models have been proposed to describe the observed trend of ion sorption on solid surfaces (sorbents); the most commonly used sorption isotherms are described below [39].

In 1926, Freundlich developed a general power equation to describe the sorption behavior of radionuclides onto different adsorbent materials [39,40]. It has the form shown, as follows:

(3) qe= Kf(Ce)1nf

where q_e_ (mg/g) is the concentration of Mo (spiked with ^99^Mo) adsorbed and C_e_ (mg/L) is the concentration of Mo remaining in the solution. K_f_ (mg^1-n^L^n^/g) and n_f_ (dimensionless) are constants unique to each combination of adsorbent and adsorbate.

Langmuir (1918) proposed an equation to describe the sorption of gases on the surface of solid sorbents. Afterward, this equation was used to describe the sorption of adsorbate onto different sorbent matrices in aqueous solutions [39,41]. This equation has the following form:

(4) $$q_{e}=\frac{n_{L}K_{L}C_{e}}{1+K_{L}C_{e}}\;$$

where q_e_ (mg/g) is the total concentration of solute adsorbed, K_L_ (L/mg) is an equilibrium constant, and n_L_ (mg/g) is the adsorption capacity.

Temkin adsorption isotherms were initially used to describe hydrogen adsorption on platinum electrodes in an acidic solution as a chemisorption process [42]. The Temkin adsorption isotherm model considers the interaction between adsorbate and adsorbent in the range of intermediate concentrations, assuming that the adsorption heat depending on temperature, varies linearly (decline) with adsorbate-adsorbent overlap degree [43]. This relationship is described in the following equation:

(5) $$q_{e}=\frac{\mathrm{RT}}{b_{T}}\ln(A_{T}C_{e})\;$$

where A_T_ is the Temkin isotherm equilibrium binding constant (L/g), b_T_ is the Temkin isotherm constant, R is the universal gas constant (8.314 J/mol∙K), and T is the temperature (K).

**Figure 3:**
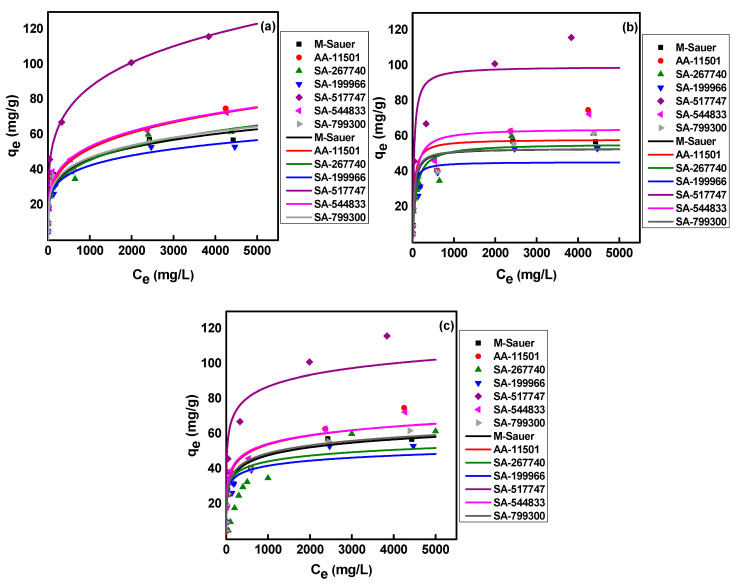
Adsorption isotherms: (**a**) Freundlich, (**b**) Langmuir, and (**c**) Temkin of CA-99Mo on different alumina adsorbents.

The measured adsorption data (C_e_ versus q_e_) of CA-^99^Mo on the alumina sorbent system were modeled using the non-linear forms of Freundlich, Langmuir, and Temkin. Adsorption isotherm models were applied, and both measured and modeled data are displayed in Figure 3. Adsorption parameters were optimized using the add-in Solver function in Microsoft Excel. Freundlich parameters (K_f_ and n_f_), Langmuir parameters (K_L_ and n_L_), Temkin parameters (A_T_ and b_T_), and the goodness of fit of the modeled lines to the experimental data (R^2^) are shown in Table 2. The regression coefficient values tabulated in Table 2 demonstrate that the Langmuir model failed to fit the equilibrium sorption isotherm of Mo(VI) on commercial alumina sorbents, as lower R^2^ values were obtained. On the contrary, good-to-excellent correlation values were obtained between the experimental results and the fitted data of the Freundlich isotherm model for all commercial alumina sorbents under investigation except for the M-Sauer and SA-799300 adsorbents, which best fit the Temkin model.

These results indicate that adsorption of CA-^99^Mo on the sorbents (AA-11501, SA-267740, SA-199966, SA-517747, and SA-544833) occurred mainly through multilayer adsorption at heterogeneous surfaces [39,44]. The Freundlich adsorption constant (n_f_) represents the adsorption intensity, for example: (i) n_f_ < 1 (a chemical process), (ii) n_f_ = 1 (linear adsorption), and (iii) n_f_ > 1 (physisorption) [44]. The n_f_ values presented in Table 2 are higher than 1, indicating that the CA-^99^Mo adsorption on commercial alumina sorbents used in this study was physisorption and favorable under the current experimental conditions. In addition, the closer the 1/n values are to 0 than to unity (ranging from 0.19 to 0.25), the more heterogeneous the surface is, implying a broad distribution of adsorption sites on the adsorbent surface [33,38]. Furthermore, Mo(VI) adsorption onto M-Sauer and SA-799300 shows a higher correlation with the Temkin model. This finding suggests that the reaction occurs in heterogeneous multilayer adsorption with a decrease in the heat of adsorption with increasing the overlap degree with Temkin constant (A_T_) 1.57 and 1.98 L g^−1^ for M-Sauer and SA-799300 adsorbents, respectively.

In order to evaluate the sorption efficiency of each material, their maximum sorption capacity for CA-^99^Mo was determined experimentally. Under our experimental conditions, a maximum sorption capacity of 50 ± 3, 66 ± 4, 64 ± 4, 52 ± 3, 148 ± 8, 72 ± 3 and 68 ± 4 mg Mo/g were reached for CA-^99^Mo by using M-Sauer, AA-11501, SA-267740, SA-199966, SA-517747, SA-544833, and SA-799300, respectively.

The obtained results reveal that out of the commercial alumina investigated in this study, SA-517747 exhibited a unique sorption profile and demonstrated a higher sorption capacity than the conventional alumina currently used in ^99^Mo/^99m^Tc generators. Therefore, it can be considered a promising sorbent for developing a ^99m^Tc generator based on LSA ^99^Mo for industrial applications.

In order to establish an efficient sorption process, the equilibrium time and the kinetic sorption parameters were investigated. Therefore, the contact time needed for a complete uptake of CA-^99^Mo onto SA-517747 was monitored. The obtained data shows a rapid and instantaneous removal of Mo from the solution, and equilibrium was established within the first minute. The results indicate that the equilibrium time was already reached at the very beginning of the sorption process. Accordingly, using the current methodology, such data cannot be modeled to sorption kinetic models.

### 2.4. Preparation of ^99^Mo/^99m^Tc Generator

**Figure 4:**
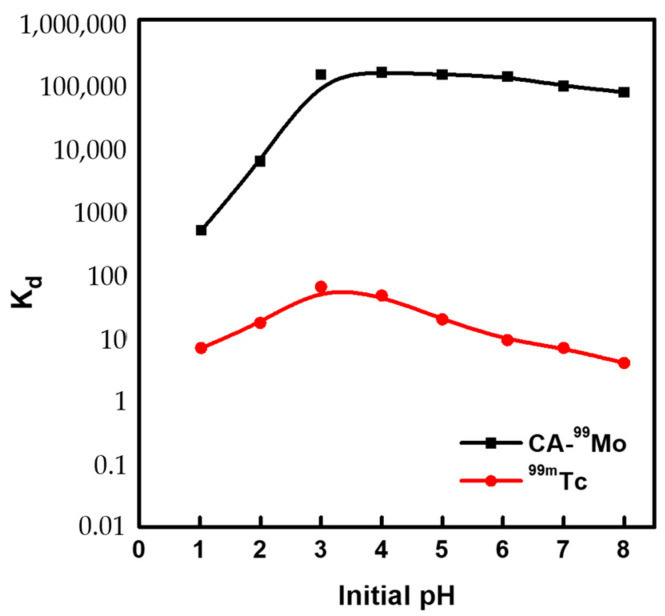
Distribution ratios (K_d_) of CA-^99^Mo and ^99m^Tc on mesoporous alumina (SA-517747).

The distribution ratio (K_d_) is a helpful indicator for investigating the selective sorption behavior of mesoporous alumina (SA-517747) for the parent ^99^Mo from aqueous solutions and the feasible elution of its daughter ^99m^Tc. Larger K_d_ values indicate that a more significant amount of ions can be retained on the sorbent material. The K_d_ data for ^99^Mo and ^99m^Tc on mesoporous alumina (SA-517747) are depicted in Figure 4. The figure shows that mesoporous alumina exhibits significant sorption affinity for CA-^99^Mo and negligible affinity for ^99m^Tc. The K_d_ of CA-^99^Mo shows high values at around pH 4, which is optimum for ^99^Mo sorption. Nonetheless, Kd values were kept consistent along the investigated pH range. To better understand this behavior, the isoelectric point (pH_IEP_) of mesoporous alumina was determined experimentally and found to be 7.1 ± 0.5. In addition, the measured equilibrium pH values of the aqueous solution did not exceed this value (Figure 1d). Therefore, optimum conditions were maintained for Mo sorption onto SA-517747 at almost all the investigated pH ranges [19,22,33].

Furthermore, the distribution ratio (K_d_) of the molybdate (MoO_4_^2−^) and the pertechnetate (^99m^TcO_4_^−^) anions on mesoporous alumina was monitored in 0.9% NaCl solution. The obtained data show high K_d_ values of CA-^99^Mo and lower K_d_ values of ^99m^Tc in 0.9% NaCl solution. The low K_d_ value of ^99m^TcO_4_^−^ in 0.9% NaCl indicates its feasible elution from the column matrix [19,22]. The ^99^Mo sorption-^99m^Tc elution mechanisms are discussed in [22,33].

**Figure 5:**
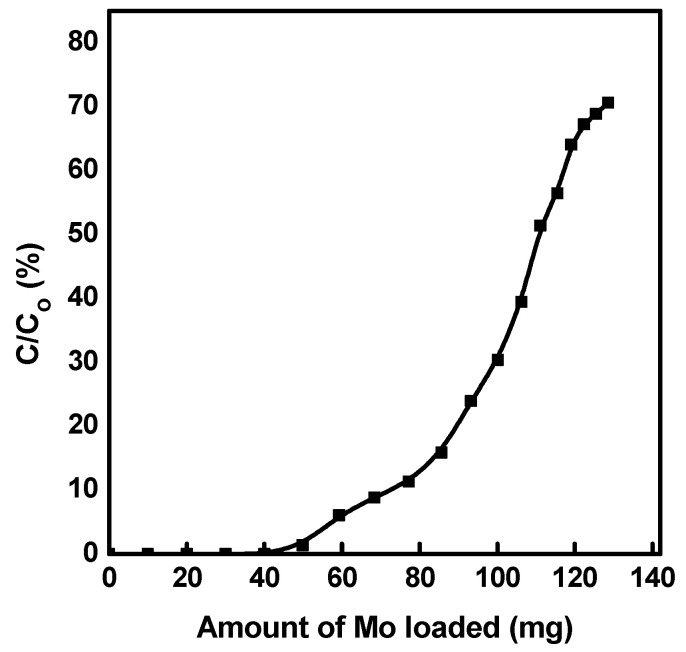
Breakthrough profile of CA-^99^Mo on mesoporous alumina (SA-517747).

Under column conditions, the dynamic capacity profile of CA-^99^Mo onto mesoporous alumina (SA-517747) was studied and is depicted in Figure 5. The figure illustrates that the breakthrough capacity reaches 44 ± 3 mg Mo/g. After reaching this value, ^99^Mo starts to appear in the effluent solution. The calculated dynamic sorption capacity at C/C_0_ = 0.5 is 108 ± 6 mg Mo per gram of sorbent material. These values are higher than the capacity of the conventional alumina (2–20 mg Mo/g of alumina) [22,24].

The data obtained from the distribution ratio (K_d_) and the dynamic sorption profile of CA-^99^Mo are promising for developing a ^99^Mo/^99m^Tc generator based on mesoporous alumina using a higher amount of activity, about 500 MBq (13.5 mCi). Then, the column was washed and conditioned for the elution of the generated ^99m^Tc by passing two consecutive solutions, namely acetate buffer solution and 0.9% saline solution, through the column. Eventually, the ^99m^Tc elution profile was further studied.

**Figure 6:**
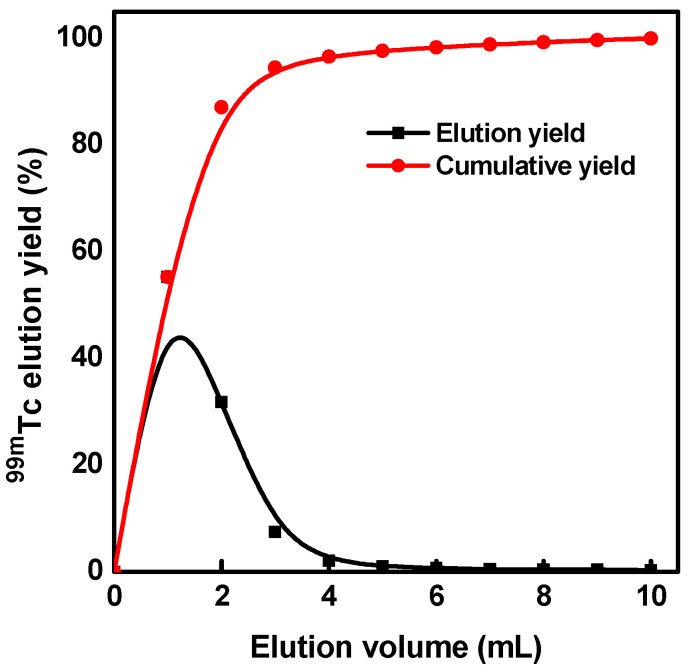
Elution profile of ^99m^Tc from mesoporous alumina-^99^Mo column.

Figure 6 shows the ^99m^Tc typical elution profile from a mesoporous alumina column. The elution process was periodically conducted using 10 mL of 0.9% NaCl solution at a 1 mL/min flow rate. It can be observed that about 95% of the ^99m^TcO_4_^−^ radioactivity is concentrated in the first 5 mL of the eluate, which indicates a sharp elution profile and that high concentrations of radioactive ^99m^TcO_4_^−^ could be obtained. Furthermore, the elution performance studies prove that the ^99m^Tc elution yield is reproducible and does not depend on the elution frequency of the generator (84 ± 0.73%) over two weeks.

**Figure 7:**
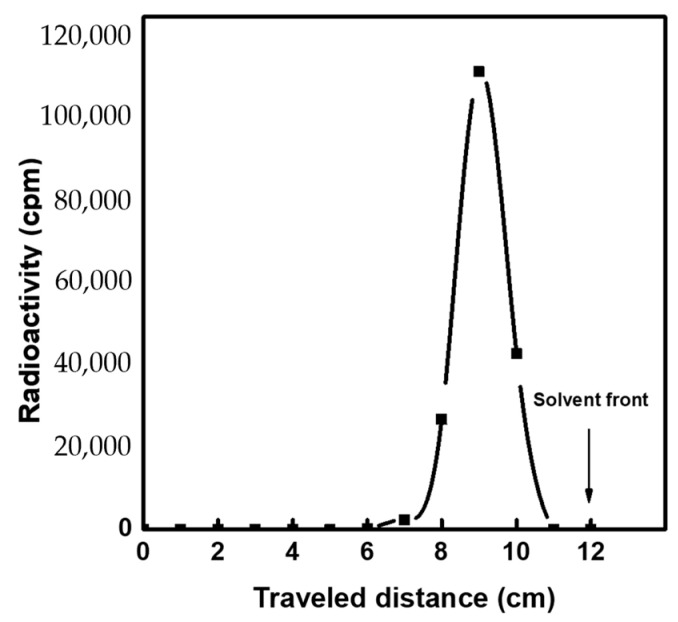
Radiochromatogram of the eluted ^99m^Tc.

In order to evaluate the effectiveness of the radiochemical separation process, the generated eluates were examined based on their radiochemical, radionuclidic, and chemical purity. To investigate the radiochemical purity, ^99m^TcO_4_^−^ species were separated using chromatography paper (Whatman No. 1) developed in 85% methanol medium. Figure 7 shows the radio-chromatogram obtained for the ^99m^Tc eluates. In all chromatograms, the radiochemical (RC) purity was >99%, and only one peak was detected at $R_{f}$ ≈ 0.8 corresponding to ^99m^TcO_4_^−^ [45,46]. This value agrees with the recommended specifications for the preparation of ^99m^Tc-labelled compounds [31,47,48].

Since ^99^Mo is the prime impurity that may interfere with the eluate, determining its impurity level is a priority. The eluate purity was evaluated using gamma-ray spectrometry (HPGe detector coupled with a multichannel analyzer). The ^99m^Tc eluate was analyzed immediately after elution and after 60 h from elution, respectively. We observed that no energy peaks corresponding to ^99^Mo were detected, and only the energy peak of ^99m^Tc (140 keV) was detected in the eluates. The absence of any ^99^Mo gamma-energy peak in the collected samples indicated that the ^99m^Tc was obtained in an adequately pure form. The analysis of the decayed samples of ^99m^Tc verified that the ^99^Mo breakthrough in the eluate was ≤0.1%. Furthermore, the decay curve of ^99m^Tc eluate was investigated, and the data show that the eluate decays with a half-life of ≈6 h, which verifies the high purity of the ^99m^Tc eluate [49,50].

The presence of chemical impurities hinders the labeling efficacy of ^99m^Tc. These impurities may originate from the column bed matrix. Therefore, ^99m^Tc eluates were chemically analyzed using ICP-MS to detect the presence of Al. The results revealed that Al concentrations were in the range of <1 µg/mL. The pH of ^99m^Tc eluates was also measured by using pH paper. The pH values were found to be in the range of 6–6.5. These values are in agreement with the recommended value for ^99m^Tc eluates [19,45,50]. Table 3 displays the elution performance data of ^99m^Tc eluates from the prepared ^99^Mo/^99m^Tc generator.

**Figure 8:**
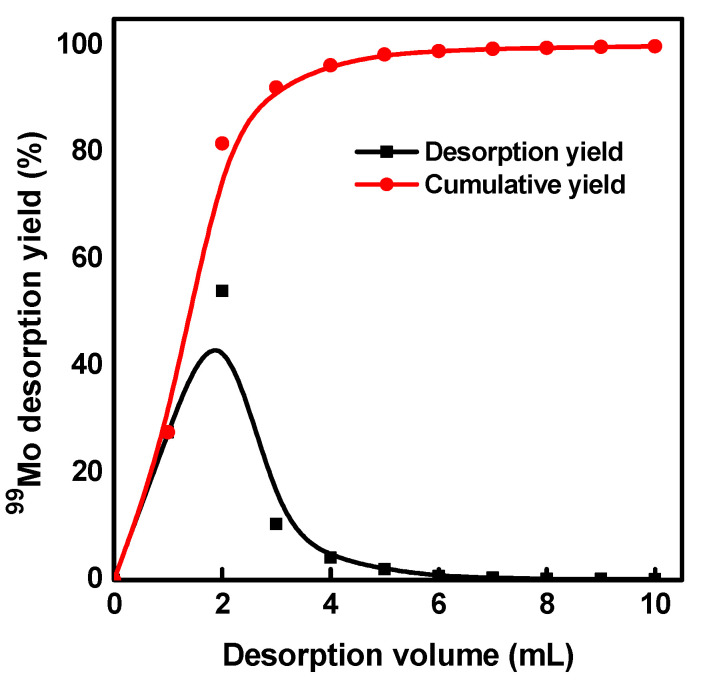
Desorption profile of ^99^Mo from the spent generator column.

Since the generator under a particular activity is no longer helpful for industrial applications, we attempted to remove the adsorbed ^99^Mo for fast and safe disposal. Figure 8 shows the desorption profile of ^99^Mo from the mesoporous alumina column. The figure shows that ^99^Mo can be desorbed under alkaline conditions. Furthermore, nearly all of the loaded ^99^Mo can be quickly recovered in the first 4–5 mL of 2 M NaOH, with a total recovery yield of >95%.

## 3. Materials and Methods

### 3.1. Materials

All chemicals were of analytical grade purity (A. R. grade) and were used without further purification. Milli-Q water was used for preparing solutions and washings. Sodium hydroxide and nitric acid were purchased from Merck, Darmstadt, Germany. The aluminum oxides were purchased from different suppliers (Table 4).

The ^99^Mo radiotracer solution was obtained by eluting 59.1 GBq fission ^99^Mo from an alumina-based ^99^Mo/^99m^Tc generator (Pertector, National Centre for Nuclear Research, Otwock, Poland) with 5 mL 1 M NaOH solution after ≈7 days from the calibration date. The total ^99^Mo radioactivity was measured with a Capintec Radioisotopes Calibrator (model CRC-55tR, Florham Park, NJ, USA). The ^99^Mo eluate solution was passed through a 0.45 micro-Millipore filter to retain any possible alumina particles. Then, the ^99^Mo solution was treated with HNO_3_ solution to attain the desired pH value.

### 3.2. Instrumentation

Radiometric identifications and measurements were carried out by using a multichannel analyzer (Inspector 2000 model, Canberra Series, Meriden, CT, USA.) coupled with a high-purity germanium coaxial detector (HPGe). Samples of constant geometry were counted at a low dead time (<5%). The radionuclide levels were determined by quantifying the 140 and 740 keV photo peaks corresponding to ^99m^Tc and ^99^Mo, respectively. A pH-meter with a microprocessor (Mettler Toledo, Seven Compact S210 model, Greifensee, Switzerland) was used to adjust the pH values. A thermostated shaking water bath (Julabo GmbH, Seelbach, Germany) was used for all batch equilibrium studies. Zeta potential (ζ) measurements were performed using a zeta-sizer Nano ZS (Malvern, UK) for isoelectric point (pH_IEP_) measurements. The chemical analyses to determine trace levels of metal contaminations were performed using inductively coupled plasma-mass spectroscopy (NexION 2000s ICP-MS, PerkinElmer, Waltham, MA, USA).

### 3.3. Static Equilibrium Studies

The batch equilibrium experiments were conducted to investigate the sorption performance of carrier-added (CA) ^99^Mo (Mo(IV) spiked with ^99^Mo) under different experimental conditions, such as solution pH, initial Mo concentration, temperature, and reaction time. In the first step, the impact of solution pH was evaluated over a broad range of pH values ranging from 1 to 8 by equilibrating 200 mg of each alumina sorbent with 20 mL of 50 mg/L CA-^99^Mo solutions. The pH value was adjusted by adding a few drops of HNO_3_ or NaOH. Each run of vials was kept at 25 ± 1 °C and shaken for 24 h in a thermally-controlled water bath shaker at a speed of 180 rpm. Then, the supernatant was decanted and centrifuged at 4000 rpm for 10 min. After that, 1 mL was pipetted and measured using a γ-ray spectrometer. Moreover, the pH of the Mo(IV) solution before and after reaching equilibrium was measured with a bench-style pH meter. In the second step, the effect of temperature on the CA-^99^Mo sorption behavior onto each sorbent was investigated at four reaction temperatures, namely 298, 313, 323 and 333 K. The CA-^99^Mo concentration was 1000 mg/L, and all other reaction parameters were kept constant. In the third step, equilibrium isotherm studies were conducted, applying the same previous experimental procedure while varying the initial molybdate concentration (50–5000 mg/L) and adjusting the initial Mo solution pH to (pH ≈ 3). Other parameters, such as reaction temperature and time, were kept at 25 ± 1 °C and 24 h, respectively. In the fourth step, the maximum adsorbent sorption capacity was determined by repeatedly equilibrating CA-^99^Mo with different alumina sorbents under optimum reaction conditions. This procedure was repeated several times until complete saturation of each sorbent material with CA-^99^Mo was achieved, and no further uptake occurred. Finally, the progress of CA-^99^Mo uptake as a function of agitation time was monitored at different time intervals for an initial Mo concentration of 50 mg/L (pH ≈ 3), using a sorbent dose of 200 mg, and the reaction temperature was adjusted to 25 ± 1 °C.

#### Data Presentation

The sorption data of CA-^99^Mo, such as uptake percent (U%), distribution coefficient (K_d_), sorption equilibrium capacity (q_e_), Mo(IV) equilibrium concentration (C_e_), and maximum sorption capacity ($q_{\max}$) were calculated according to the following equations:

(6) $$\;U\;\%\;=\frac{A_{o}-{\;A}_{e}}{A_{o}}\times 100$$

(7) $$\;\;\;K_{d\;}=\frac{A_{o}-{\;A}_{e}}{A_{e}}\times \frac{v_{1}}{m}\;\;\;\;\;\;\;\;\;\;\left(\mathrm{mL}/g\right)$$

(8) $$q_{e}=\frac{A_{o}-{\;A}_{e}}{A_{o}}\times {\;C}_{o}\times \frac{v}{m}\;\;\;\;\left(\mathrm{mg}/g\right)\;$$

(9) $$C_{e}={\;A}_{o\;}-\left(A_{o}\times \frac{U\%}{100}\right)\;\;\;\;\;\;\;\;\;\;\;\;\left(\mathrm{mg}/L\right)$$

(10) $$q_{\max\;}=\frac{\sum^{\;}U\%}{100}\times {\;C}_{o}\times \frac{v}{m}(\mathrm{mg}/g)$$

where A_o_ and A_e_ are the initial and equilibrium ^99^Mo radioactivity (counts/min), respectively. v_1_ (mL) and v (L) are the liquid phase volume. m is the sorbent weight (g) and $C_{o}$ is the equilibrium Mo(IV) concentration in (mg/L).

### 3.4. Application of Mesoporous Alumina in Preparing a ^99^Mo/^99m^Tc Generator

To evaluate the dynamic sorption capacity of mesoporous alumina for CA-^99^Mo under column conditions, we packed 1 g of the sorbent material in a column of dimensions (12 cm length × 0.4 cm i.d.) with a sintered disc at the bottom. Then, the column matrix was treated with 10^−3^ M HNO_3_. Subsequently, sodium molybdate solution (5 mg Mo/mL), spiked with 370 kBq (10 µCi) of ^99^Mo tracer, was passed through the columns at a flow rate of 0.25 mL/min. In order to monitor the adsorption pattern, 2 mL of the mother feed solution was kept as a reference (C_o_). Likewise, the effluent volume was collected in fractions of 2 mL aliquots (C). Then, the count rate ratio of each fraction to the count rate of the mother feed solution was determined by measuring the 740 keV γ-ray peak of ^99^Mo in a HPGe detector. Eventually, the capacity was calculated by using the following equation:

(11) $$\mathrm{Capacity}\;=\frac{C_{o}\times {\;V}_{50\%}}{m}\;\mathrm{mmol}/g$$

where $C_{o}$ is the initial Mo ion concentration in its feeding solution, $V_{50\%}$ is the effluent volume (mL) at C/C_0_ = 0.5, and m (g) is the weight of the column matrix.

To design a ^99^Mo/^99m^Tc generator, a column with 1 g of mesoporous alumina (SA-517747) was packed and conditioned with HNO_3_. Then, it was loaded with 130 mg Mo spiked with 500 MBq of ^99^Mo (pH ≈ 3) by using the previously mentioned protocol. Subsequently, the column was washed with 50 mL of acetate buffer solution and 100 mL of 0.9% saline solution. Finally, the column was left for about 24 h before the ^99m^Tc elution.

### 3.5. Elution Performance of ^99m^Tc Eluate

In order to investigate the ^99m^Tc elution performance, the generator was eluted with 0.9% NaCl solution at a 1 mL/min flow rate at different time slots. The eluates were collected in equal fractions (1 mL each) and immediately analyzed. In order to identify the contribution of foreign radionuclidic contaminants in the ^99m^Tc solution, the eluates were radiometrically analyzed immediately after elution and subsequently after 60 h. Additionally, the radionuclidic purity of ^99m^Tc eluate was studied by following its radioactive decay.

The radiochemical purity of the eluted ^99m^Tc (percentage of ^99m^TcO_4_^−^ to the total activity of the eluate) was determined by ascending paper chromatography using Whatman no. 1 paper and a mixture of (85% methanol + 15% H_2_O) as developing solvent. The radioactivity distributions were monitored using a γ-ray spectrometer to determine R_f_. The measured radioactivity was plotted as a function of the traveled distance from the starting line. The $R_{f}$ value was calculated according to the following equation:

(12) $$R_{f}=\frac{\mathrm{The}\;\mathrm{distance}\;\left(\mathrm{Cm}\right)\mathrm{from}\;\mathrm{the}\;\mathrm{starting}\;\mathrm{line}\;\mathrm{to}\;\mathrm{the}\;\mathrm{radioactivity}\;\mathrm{peak}\;\mathrm{position}}{\mathrm{The}\;\mathrm{distance}\;\left(\mathrm{Cm}\right)\mathrm{from}\;\mathrm{the}\;\mathrm{starting}\;\mathrm{line}\;\mathrm{to}\;\mathrm{the}\;\mathrm{solvent}\;\mathrm{front}}\;$$

Any possible impurities of aluminum were determined in the ^99m^Tc eluates originating from the column matrix. The aluminum level was measured by using inductively coupled plasma-mass spectroscopy (ICP-MS). All tests were performed after complete decay of ^99^Mo and ^99m^Tc in the eluates.

### 3.6. Recovery of ^99^Mo from the Spent Generator

The exhausted generator was rinsed with 20 mL of 0.9% saline. Then, Mo was desorbed using a 2 M NaOH solution at a 0.5 mL/min flow rate. The desorbed solution was recovered in fractions of 1 mL each. Subsequently, each fraction was measured, and the total recovery yield of ^99^Mo was investigated.

## 4. Summary and Conclusions

The objective of this paper was to investigate the feasibility of using commercial alumina incorporated with LSA ^99^Mo to develop a useful ^99^Mo/^99m^Tc generator for industrial radiotracer applications. From the research conducted in this study, we found that molybdenum is selectively adsorbed on a mesoporous alumina (SA-517747) column, and the sorbent exhibits a high equilibrium and dynamic sorption capacities for LSA ^99^Mo (148 ± 8 and 108 ± 6 mg Mo/g). Moreover, ^99m^Tc could be eluted with high yield and adequate chemical, radiochemical, and radionuclidic purity. The available specific activity of LSA ^99^Mo can reach 5 Ci/g Mo. Therefore, based on our findings, it is possible to build a ^99m^Tc generator of 18.5 GBq (500 mCi) at calibration time using 1 g of mesoporous alumina (SA-517747). Consequently, mesoporous alumina is a viable option for developing a ^99m^Tc generator based on ^99^Mo of LSA. This method provides an efficient and cost-effective way to supply onsite ^99m^Tc for radiotracer applications independent of fission-produced ^99^Mo technology.

## Figures and Tables

**Table 1:** Thermodynamic parameters for the sorption of CA-^99^Mo on different alumina adsorbents.

Adsorbent	Temperature (K)	ΔG° (kJ/mol)	ΔH° (kJ/mol)	ΔS° (kJ/mol∙K)
M-Sauer	298	−10.508	−8.934	0.005
	313	−10.588		
	323	−10.640		
	333	−10.693		
AA-11501	298	−10.602	−7.225	0.011
	313	−10.772		
	323	−10.885		
	333	−10.998		
SA-267740	298	−9.563	5.877	0.052
	313	−10.340		
	323	−10.859		
	333	−11.377		
SA-199966	298	−9.503	9.208	0.063
	313	−10.444		
	323	−11.072		
	333	−11.700		
SA-517747	298	−10.405	22.132	0.109
	313	−12.043		
	323	−13.135		
	333	−14.227		
SA-544833	298	−10.975	4.089	0.051
	313	−11.733		
	323	−12.238		
	333	−12.744		
SA-799300	298	−9.787	7.756	0.059
	313	−10.670		
	323	−11.258		
	333	−11.847		

**Table 2:** Isotherm parameters calculations for the adsorption of carrier-added ^99^Mo on different alumina adsorbents.

Isotherm Model	Parameter	M-Sauer	AA-11501	SA-267740	SA-199966	SA-517747	SA-544833	SA-799300
Freundlich	n_f_	5.046	4.332	4.484	5.485	4.637	4.561	5.066
	K_f_ (mg^1−n^L^n^/g)	11,698.05	10,581.47	9779.30	12,073.05	19,643.67	11,720.94	12,051.36
	R^2^	0.93	0.96	0.97	0.96	0.99	0.98	0.95
Langmuir	n_L_ (mg/g)	53,211.56	58,393.90	55,956.60	45,611.11	99,583.12	64,505.84	53,278.67
	K_L_ (L/mg)	0.0234	0.0253	0.0115	0.0350	0.0290	0.0152	0.0258
	R^2^	0.94	0.8	0.89	0.83	0.9	0.94	0.89
Temkin	A_T_ (L/g)	1.5688	1.48864	11.7524	22.3752	9.66192	2.26332	1.983
	b_T_ (KJ/mol)	0.00428	0.00376	0.00588	0.00665	0.00293	0.00395	0.00431
	R^2^	0.97	0.92	0.81	0.88	0.91	0.95	0.98

**Table 3:** The elution yield and quality control data of ^99m^Tc eluates.

Elution No.	^99m^Tc Growth Period, h	Quality Control of the Eluted ^99m^Tc			
		^99m^Tc Elution Yield, %	R.C. Purity, (^99m^TcO_4_^−^, %)	Chemical Purity	
				Al Content, µg/mL	pH Value
1	24	84.6	>99	<1	6
2	24	84.5			6
3	24	85.0			6
4	24	84.6			6
5	24	83.7			6
6	24	85.0			6
7	24	83.8			6
8	24	83.5			6
9	24	83.4			6.5
10	48	84.0			6.5
12	24	82.9			6.5

**Table 4:** Description of the analyzed alumina *.

No.	Name	Supplier	Description	Particle Size	Surface Area	pH
1	M-Neutral	Merck	Activity stage I, neutral	63–200 µm	120 m^2^/g	6.8–7.8
2	M-Sauer	Merck	Activity stage I, acidic	63–200 µm	120 m^2^/g	3.5–4.5
3	AA-11501	Alfa Aesar	Activated, acidic	60 mesh	150 m^2^/g	4.5 ± 0.5
4	AA-46064	Alfa Aesar	Activated, acidic	50–200 µm	N.A	N.A
5	AA-11502	Alfa Aesar	Activated, neutral	60 mesh	150 m^2^/g	N.A
6	SA-267740	Sigma-Aldrich	Weakly acidic	150 mesh	155 m^2^/g	6.0
7	SA-199966	Sigma-Aldrich	Activated, acidic	50–300 mesh	155 m^2^/g	4.5 ± 0.5
8	SA-769290	Sigma-Aldrich	Ultra-dry	63 µm	120–190 m^2^/g	N.A
9	SA-199974	Sigma-Aldrich	Activated, neutral	40–160 µm	205 m^2^/g	7.0 ± 0.5
10	SA-517747	Sigma-Aldrich	Nano mesoporous (Pore size = 3.8 nm)	N.A	N.A	N.A
11	SA-544833	Sigma-Aldrich	Nanopowder	<50 nm	>40 m^2^/g	N.A
12	SA-799300	Sigma-Aldrich	Activated, acidic	50–300 mesh	155 m^2^/g	4.5 ± 0.5

*: The information was provided by the supplier.

## Data Availability

Data are contained within the article.

## Sample Availability

The alumina adsorbents were purchased from different commercial suppliers.

## Abbreviations

AA	Alfa Aesar
M	Merk
N.A	Not Available
SA	Sigma-Aldrich
